# Competencies required for the performance of primary health care managers: a systematic review

**DOI:** 10.1590/0102-311XEN092624

**Published:** 2025-01-27

**Authors:** Katherine Soto-Schulz, Raúl Herrera-Echenique, Rodrigo Brito-Díaz, Nuria Pérez-Romero

**Affiliations:** 1 Facultad de Ciencias de la Rehabilitación, Universidad Andrés Bello, Santiago de Chile, Chile.; 2 Programa de Doctorado en Derecho y Administración de Empresas, Universidad de Lleida, Lleida, España.; 3 Facultad de Economía y Negocios, Universidad Andrés Bello, Santiago de Chile, Chile.; 4 Instituto de la Visión, Santiago de Chile, Chile.

**Keywords:** Primary Health Care, Health Services, Health Administration, Health Personnel, Atención Primaria de Salud, Servicios de Salud, Administración en Salud, Personal de Salud, Atenção Primária à Saúde, Serviços de Saúde, Administração em Saúde, Pessoal de Saúde

## Abstract

This study aimed to identify the competencies required by primary health care managers for the effective performance of their functions. A systematic review was conducted according to PRISMA, in the databases PubMed, Scopus, Web of Science, and CINAHL, up to May 2023, in the last 10 years. The inclusion criteria were quantitative, qualitative, or mixed studies that evaluated the competencies required for primary health care managers and published in English, Spanish, or Portuguese. Methodological quality was assessed using the *Mixed Methods Appraisal Tool*. This article identified 171 studies, including six to the analysis. The importance of leadership, teamwork, and communication was highlighted. Furthermore, the need for disciplinary training in the health area, knowledge in administration, and use of management indicators, as well as an autonomous and flexible attitude to challenges were highlighted. The evaluation of methodological quality showed an overall good performance, except for some studies that do not report sufficient information to determine sample representativeness. Primary health care managers must possess specific competencies to effectively perform their roles, given the relevance of primary care in each country’s health system. This study provides a general framework of the required competencies for managerial responsibilities in this area. However, it is necessary to consider the particularities and local contexts of each center to develop managerial profiles adapted to their specific needs.

## Resumen

El objetivo de este estudio fue identificar las competencias necesarias para que los gestores de atención primaria de salud puedan desempeñar eficazmente sus funciones. La revisión sistemática se realizó de acuerdo con las recomendaciones de PRISMA en las bases de datos PubMed, Scopus, Web of Science y CINAHL, considerando publicaciones de los últimos 10 años hasta mayo de 2023. Los criterios de inclusión fueron los siguientes: estudios cuantitativos, cualitativos o mixtos que evaluaron las competencias requeridas para los gestores de atención primaria de salud y que estaban publicados en inglés, español o portugués. La calidad metodológica se evaluó utilizando el *Instrumento de Evaluación de Métodos Mixtos*. Se encontraron 171 estudios; y seis se agregaron al análisis. Se destacaron la importancia del liderazgo, del trabajo en equipo y de la comunicación. Es necesaria una formación disciplinaria en el área de salud, además de conocimiento en la administración y gestión de indicadores, así como una actitud autónoma y flexible frente a los desafíos que se presentan en su trabajo. La evaluación de la calidad metodológica presentó, en general, un buen desempeño, a excepción de algunos estudios que no aportan información suficiente para determinar la representatividad de las muestras. Los gestores de atención primaria de salud deben contar con competencias específicas para desempeñar sus funciones de manera efectiva, dada la relevancia del nivel primario en los sistemas de salud de cada país. Este estudio proporciona una visión general de las competencias requeridas para las responsabilidades gerenciales en esta área; sin embargo, es necesario considerar las particularidades y contextos locales de cada centro para desarrollar perfiles gerenciales adaptados a sus necesidades específicas.

## Resumo

O objetivo foi identificar as competências necessárias para que os gestores de atenção primária à saúde desempenhem efetivamente suas funções. A revisão sistemática foi realizada de acordo com as recomendações PRISMA nas bases de dados PubMed, Scopus, Web of Science e CINAHL, considerando publicações dos últimos 10 anos até maio de 2023. Os critérios de inclusão foram: estudos quantitativos, qualitativos ou mistos que avaliaram as competências necessárias para gestores de atenção primária à saúde e publicados em inglês, espanhol ou português. A qualidade metodológica foi avaliada utilizando o *Instrumento de Avaliação de Métodos Mistos*. Foram identificados 171 estudos, sendo que seis foram analisados. Destacou-se a importância da liderança, do trabalho em equipe e da comunicação. Além da necessidade de formação disciplinar na área da saúde, foi ressaltado o conhecimento em administração e gestão de indicadores, assim como uma atitude autônoma e flexível diante dos desafios que surgem em seu trabalho. A avaliação da qualidade metodológica apresentou, de modo geral, um bom desempenho, exceto por alguns estudos que não fornecem informações suficientes para determinar a representatividade das amostras. Os gestores de atenção primária à saúde devem possuir competências específicas para desempenhar suas funções de forma eficaz, dada a relevância do nível primário nos sistemas de saúde de cada país. Este estudo oferece um quadro geral das competências necessárias para as responsabilidades gerenciais nesta área; no entanto, é necessário considerar as particularidades e os contextos locais de cada centro para desenvolver perfis gerenciais adaptados às suas necessidades específicas.

## Introduction

The *Declaration of Alma-Ata* in 1978 marked an important milestone for health development worldwide, highlighting the need for governments to formulate policies and plans that can incorporate primary health care (PHC) as part of a comprehensive health system, promoting access and participation of people in planning their health care system ^1^. According to the World Health Organization (WHO), PHC is a key factor for the universal and sustainable coverage of a health system ^2^ ^,^ ^3^ ^,^ ^4^, being one of the most effective strategies to respond to current health challenges. These challenges include problems derived from unhealthy lifestyles, acceleration of unplanned urbanization, or demographic aging that leads to an increase in noncommunicable diseases, increasing the demands to be met by the health system ^5^. Forty years after Alma-Ata, the Pan American Health Organization (PAHO) convened the Fifth Regional Forum entitled *Universal Health in the 21st Century: 40 years after Alma-Ata*, aiming at developing recommendations for people’s right to health to be effective. Among the recommendations that emerged from this forum, the need to ensure an institutional model that guarantees people’s right to health and to strengthen the PHC-based care model stand out, incorporating the rational use of resources, the development of a financing model that safeguards the system’s equity, coverage, and sustainability. The valuation of Human Resources also stands out ^6^, in which adequate planning and development is crucial for the effective implementation of a comprehensive health system and the PHC-based care model ^7^ ^,^ ^8^.

The challenges derived from PHC are multiple, requiring trained and competent professionals to provide an effective response to the health demands of the population; the definition of policies and plans that ensure the incorporation of professionals prepared for the changes that arise in health systems, in addition to the generation of adequate and challenging work environments that promote the commitment of workers to the institutional mission constitute one of the most relevant challenges in terms of Human Resources ^9^. To achieve the best performance expected from professionals, the concept of competency emerges. Such concept comprises knowledge, skills, and attitudes required for a successful performance in a specific activity ^10^, playing a fundamental role in achieving the objectives of PHC teams ^11^.

The study of competencies in PHC managers has evolved significantly in recent decades, reflecting the growing recognition of the critical role these leaders play in the health system. Recent literature emphasizes the need for a comprehensive understanding of both the managerial and clinical competencies required for effective leadership in PHC settings ^12^. Studies have identified key competencies such as strategic vision, interpersonal communication, and the ability to drive organizational change as essential traits for PHC leaders. Moreover, the importance of continuous professional development and training tailored to the unique challenges faced by PHC managers has been highlighted as a priority in current research ^13^ ^,^ ^14^.

The essential elements that constitute PHC-based health systems are a concrete orientation of the competencies that teams should have to respond to the situations and contexts defined in them. To achieve an adequate functioning of these teams, committed managers are required, with knowledge in Health and Administration, a vision of the context in which they work and a range of managerial skills that allow them to lead their team effectively to achieve goals defined for the unit ^15^. Leadership is an essential skill in any manager, however, there is still a need to develop a deeper knowledge on leadership skills for the implementation of an integrated health system ^16^.

Given the relevance of having suitable professionals to lead the implementation of a PHC-based health system, in addition to the growing challenges experienced by the Health sector, which must be responded efficiently and effectively, this systematic review aims to identify the necessary competencies for the optimal performance of PHC managers.

## Material and methods

A systematic literature review was performed following the guidelines of the *Preferred Reporting Items for Systematic reviews and Meta-Analyses* (PRISMA) ^17^. The review protocol was previously registered and can be found at: https://inplasy.com/inplasy-2023-9-0013/.

### Search strategy

The systematic search for articles was conducted in the MEDLINE (PubMed), Scopus, Web of Science and CINAHL databases with a deadline of May 2023. Box 1 shows the search terms. The studies were exported to Rayyan.ai (https://www.rayyan.ai/) for identification of duplicates and document selection ^18^.

**Box 1:** Search strategy in each database.

DATABASE	SEARCH STRATEGY
PubMed	(((((““personnel management”“) OR (““social skills”“)) OR (“professional competence”)) OR (“management competencies”)) OR (“managerial skills”)) AND ((((((“executives”) OR (“health managers”)) OR (“managers”)) AND (“Primary Health Care”)) OR (“Primary Care”)) OR (“health care primary”)) Filters: in the last 10 years
Scopus	((“personnel management”) OR (“social skills”) OR TITLE (“professional competence”) OR (“management competencies”) OR (“managerial skills”)) AND (((“executives”) OR (“health managers”) OR (“managers”)) AND ((“Primary Health Care”) OR TITLE (“Primary Care”) OR (“health care primary”)) AND PUBYEAR > 2013 AND PUBYEAR < 2023
Web of Science	1: “personnel management” 2: “social skills” 3: “professional competence” 4: “management competencies” 5: “managerial skills” 6: “executives” 7: “health managers” 8: “managers” 9: “Primary Health Care” 10: “Primary Care” 11: “health care primary” 12: #1 OR #2 OR #3 OR #4 OR #5 13: #6 OR #7 OR #8 14: #9 OR #10 OR #11 15: #12 AND #13 AND #14 and 2013 or 2014 or 2015 or 2016 or 2017 or 2018 or 2019 or 2020 or 2021 or 2022 or 2023 (Publication Years)
CINAHL	(“personnel management” OR “social skills” OR “professional competence” OR “management competencies” OR “managerial skills”) AND (“executives” OR “health managers” OR “managers”) AND (“Primary Health Care” OR “Primary Care” OR “health care primary”) Filters: in the last 10 years

### Selection criteria

The selection criteria for studies were: (i) primary studies of quantitative, qualitative or mixed type; (ii) assessing the competencies required by PHC managers for their performance; and (iii) published in the last 10 years.

### Study selection

Two authors independently evaluated the identified studies, first reviewing them by title and abstract, and then examining the remaining studies in full. Discrepancies were resolved by a third reviewer. Reasons for exclusion were recorded for all studies.

### Extraction and analysis

Two authors performed a qualitative synthesis of the selected studies that considered two data extraction tables; the first included information related to the characteristics of the sample of each study, including: (i) author; (ii) number of participants; (iii) sex; (iv) training; and (v) years of experience. The second table, which reflects the competencies required by PHC managers, considered: (i) author; (ii) country; (iii) design; (iv) knowledge; (v) skills; and (vi) attitudes. The information was recorded in an Excel spreadsheet (https://products.office.com/) in both cases. To evaluate methodological quality, two authors independently applied the *Mixed Methods Appraisal Tool* (MMAT), which enables the critical evaluation of systematic reviews of mixed studies, i.e., reviews that include qualitative, quantitative, and mixed methods studies ^19^.

## Results

**Figure 1:**
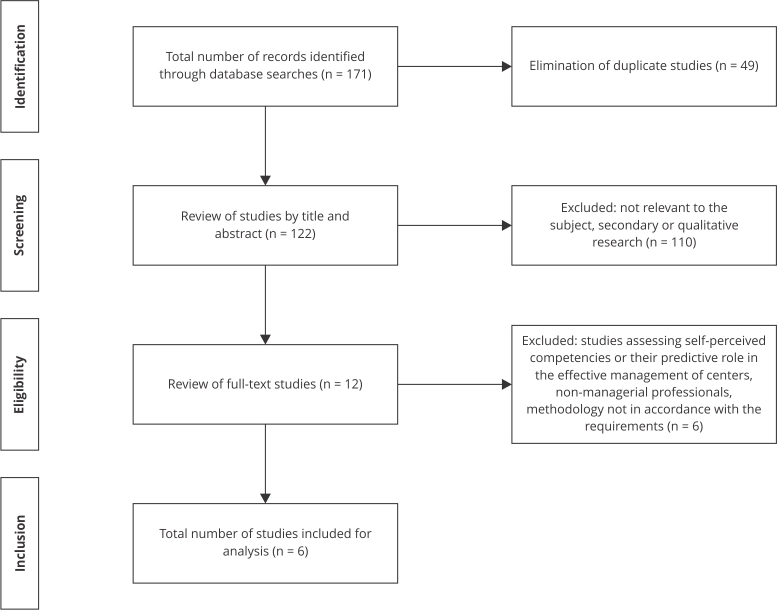
PRISMA flowchart of the selection process.

After reviewing the four databases, 171 studies were identified. After eliminating duplicates, 122 records were obtained. Once examined by title and abstract, 12 studies were read in full, eliminating 110 records. The main causes of exclusion at this stage were nonattendance to the central theme of this review or secondary studies. Finally, only six studies met the selection criteria and were included in this review ^20^ ^,^ ^21^ ^,^ ^22^ ^,^ ^23^ ^,^ ^24^ ^,^ ^25^, the remaining six were excluded because the sample included non-managerial PHC professionals, focused on the level of self-perceived competencies without informing the competency they actually required, evaluated competencies as predictors of effective health center management, or their methodology was not in accordance with the inclusion criteria ^26^ ^,^ ^27^ ^,^ ^28^ ^,^ ^29^ ^,^ ^30^ ^,^ ^31^. Figure 1 details the complete study selection process.

### Characteristics of the included studies

Of the six studies selected, three of them have a qualitative methodological design, two correspond to descriptive quantitative studies, and one to a prospective design with Delphi methodology. Regarding the country where they were conducted, four correspond to Brazil, one to Serbia, and one to Bhutan. Overall, the sample comprises PHC managers, with work experience ranging from one to 30 years. There is a predominance of women among primary care managers and different academic background. Table 1 shows the details of the sample characterization.

**Table 1:** Characteristics of the sample of selected studies.

Study (year)	Participants	Sex	Education	Years of experience
Fernandes & Cordeiro ^20^ (2019)	10	Women: 80% Men: 20%	Graduate: 100%	> 5: 100%
Farah et al. ^21^ (2017)	16	Women: 93.75% Men: 6.25%	Master’ degree: 25% Diploma: 75%	6-30: 100%
Dorji et al. ^22^ (2019)	339	Women: 34.4% Men: 64.6%	Graduate: 3.3% Bachelor: 25.4% Diploma: 3.0% Certificate: 68.3%	> 15: 45.4% 11-15: 10.3% 6-10: 17.4% 1-5: 26.8%
Dikic et al. ^23^ (2020)	106	Women: 67.9% Men: 32.1%	Graduate: 50.9% University: 34.9% Technical training: 11.3%	20 or more: 39.6% Managerial function > 1: 54.8%
Silva et al. ^24^ (2022)	8	Women: 87.5% Men: 12.5%	Secondary education: 75% High school: 25%	1-2: 37.5% 4-5: 37.5% > 5: 25.0%
André et al. ^25^ (2013)	10	NI	NI	NI

NI: unidentified.

### Methodological quality

After the application of the MMAT ^19^, it was observed that three of the six studies achieved the maximum score in each of the five items evaluated, they correspond to qualitative designs. The remaining three studies present scores ranging from two to four points. Note that the lack of detailed information by the authors affects the studies’ score in items related to the representativeness of the sample regarding target population and appropriate choice of measures. Table 2 shows the score obtained by each study.

**Table 2:** Results of methodological quality assessment with the *Mixed Methods Appraisal Tool* (MMAT).

Study	MMAT score
Fernandes & Cordeiro ^20^	5
Farah et al. ^21^	5
Dorji et al. ^22^	4
Dikic et al. ^23^	2
Silva et al. ^24^	5
André et al. ^25^	3

### Competencies required in PHC managers

According to the definition of competency, which includes the knowledge, skills and attitudes necessary to conduct a specific task or activity ^10^, training in the field of Health is identified as a necessary knowledge for managers to adequately perform their role ^24^ ^,^ ^25^; they also highlight the need to have knowledge related to the health care network, work flows, information management, and use of management indicators ^20^ ^,^ ^24^. Farah et al. ^21^ emphasize the need to have previous knowledge in Primary Care and Human Resources; the importance of an Administration background is also emphasized ^20^.

Regarding skills, leadership emerges as the most mentioned one, being identified in five studies ^20^ ^,^ ^22^ ^,^ ^23^ ^,^ ^24^ ^,^ ^25^; teamwork (the ability to form or perform adequately in a team) is identified by four studies ^20^ ^,^ ^21^ ^,^ ^23^ ^,^ ^25^, as well as the ability to plan ^20^ ^,^ ^21^ ^,^ ^23^ ^,^ ^25^. Skills related to communication or listening also acquire a relevant role ^21^ ^,^ ^22^ ^,^ ^23^ ^,^ ^25^, as well as recognizing skills in other team members ^20^ ^,^ ^21^ ^,^ ^24^. Conflict management ^20^ ^,^ ^24^, negotiation skills ^20^ ^,^ ^24^, delegating tasks ^20^ ^,^ ^21^, decision-making ^20^ ^,^ ^21^ and change management ^22^ ^,^ ^25^ are also mentioned by the authors.

Finally, for the attitudes required for PHC managers, there is an agreement between two authors on aspects related to the capacity for autonomy and flexibility ^20^ ^,^ ^21^; other attitudes also emerge, such as empathy and proactivity ^24^, as well as motivation ^21^, acting ethically, and social responsibility ^25^. Box 2 shows the complete detail of the competencies identified for each study.

**Box 2:** Description of the competencies required by primary health care managers.

STUDY	COUNTRY	DESIGN	KNOWLEDGE	SKILL	ATTITUDE
Fernandes & Cordeiro ^20^	Brazil	Qualitative, descriptive, and exploratory (focus group and Bardin content analysis)	Knowledge of the health care network and workflows; Information management and use of management indicators; Training in Administration; Management of supplies and materials	Leadership; Teamwork; Planning, monitoring and evaluation; Recognizing skills in others; Conflict management; Negotiation; Delegating tasks; Decision making; Articulate	Autonomy; Flexibility; Resilience; Neutrality; Coping; Creativity
Farah et al. ^21^	Brazil	Qualitative and descriptive (semistructured interview and dialectical hermeneutics)	Knowledge of guidelines and protocols; Knowledge in primary care and human resources	Teamwork; Planning; Listening skills; Recognizing skills in others; Delegating tasks; Decision-making; Persuasiveness	Autonomy; Flexibility; Motivation; Adaptation
Dorji et al. ^22^	Bhutan	Quantitative and cross-sectional (self-administered questionnaires)		Leadership; Communication; Relationship building; Change management; Analytical thinking; Innovative thinking	Professionalism
Dikic et al. ^23^	Serbia	Quantitative and cross-sectional (U.S. Centers for Disease and Control and Prevention questionnaire)		Leadership; Team building; Planning and prioritization; Communication; Problem solving; Evaluating performance	
Silva et al. ^24^	Brazil	Qualitative (semi-structured interviews)	Training in the Health area; Knowledge of processes and workflows; Knowledge of unit objectives, indicators, and goals	Leadership; Recognizing skills in others	Active and decisive stance in the face of demands; Proactivity; Empathy; Professional self-fulfillment
André et al. ^25^	Brazil	Prospective study (Delphi method)	Bachelor’s degree in Health; Specialization in Health Services	Leadership; Working with and developing teams; Planning, monitoring and evaluating; Effective communication; Conflict management; Negotiation; Change management; Systemic and comprehensive long-term vision	Acting with ethics and social responsibility

## Discussion

This review analyzed the available evidence related to competencies primary health care managers needed to effectively perform their work, considering the important role of PHC and its constant challenges to respond effectively and efficiently to the population’s health demands.

Regarding the knowledge required to manage PHC centers, authors point out that training in the Health area is essential for their performance via specific courses ^24^, a degree ^25^, or to know the evolution of medical and management practices ^24^. These background options should aid to informed and effective decision-making in the management of PHC units ^25^. This was also shown in a previous study in Chile that characterized the managerial profile in PHC, in which the 46.3% of the respondents mentioned having a master’s degree is important to hold managerial positions in PHC ^32^. However, there are difficulties such as lack of economic resources, low training offer, and lack of time and motivation to access this level of improvement ^32^. Fernandes & Cordeiro ^20^ pointed out that training in Administration is important for PHC managers because it helps them to develop the managerial competencies necessary for the effective performance of their functions. This could enable them to acquire technical knowledge in areas such as resource management, planning, coordination, monitoring, and evaluation of health actions. Nevertheless, Health Administration constitutes an essential element of Health Economics, providing analysis and practical instruments to achieve efficiency in health systems ^33^. Fernandes & Cordeiro ^20^ and Silva et al. ^24^ indicate the need for managers to have knowledge regarding management and use of management indicators. This becomes relevant at all levels of care, since the information they provide enables informed decision-making and continuous improvement of processes ^34^. The same authors also emphasize the need to have knowledge of workflows and health care network, which is complemented by Farah et al. ^21^, who indicate a manager should have knowledge of Human Resources and primary care operations.

Regarding the skills required for PHC managers, leadership, which can be defined as the ability to guide others toward a common goal ^35^, was the most identified ^20^ ^,^ ^22^ ^,^ ^23^ ^,^ ^24^ ^,^ ^25^. Possible reasons for such finding are based on their role in influencing and driving teamwork to achieve established goals and to obtain the necessary information to manage ^20^, or in promoting the intersectoral actions necessary to respond to the health requirements of the population ^11^. Moreover, adequate leadership not only improves the quality of care, but also results in greater satisfaction, both for patient and staff, while increasing efficiency ^30^. Other skills linked to leadership from the selected studies are negotiation skills ^20^ ^,^ ^25^, decision-making ^20^ ^,^ ^21^, change management ^22^ ^,^ ^25^, persuasion skills ^21^, relationship building ^22^, and the ability to delegate ^20^ ^,^ ^21^. This last skill becomes relevant in PHC contexts, in which effective delegation of functions or tasks is essential for proper functioning of teams and implementation of user-centered models ^36^.

Training and teamwork also acquire relevance within the competencies required for PHC managers ^20^ ^,^ ^21^ ^,^ ^23^ ^,^ ^25^. Farah et al. ^21^ point out that teamwork in primary care is collective, interdependent, and established by continuous and intense relationships, in which conflicts may be common, needing the figure of a leader to overcome challenges and safeguard the working relationships of the team. Dikic et al. ^23^ conclude that team building is a crucial competency for the performance of managers, who consider themselves highly competent in this area. This is corroborated by authors who identify this skill as necessary to meet goals in various healthcare contexts, emphasizing the need to train teams to successfully develop this skill ^37^ ^,^ ^38^. Acknowledging skills are also identified as a key competency for PHC managers ^20^ ^,^ ^21^ ^,^ ^24^. In healthcare settings, acknowledging and promoting the development of each team member’s own skills is an important element for creating high-performance teams, more effectively achieving the clinical and financial objectives of the unit ^39^.

Communication and listening skills are also identified as key competencies for the professional practice of PHC managers ^21^ ^,^ ^22^ ^,^ ^23^ ^,^ ^25^; emphasizing the importance of listening to the team and providing space to raise ideas and create participatory environments ^21^. Previous literature also indicate that communication is a key skill for the structural function of teamwork, as defined in the PHC Team Competency Matrix ^11^. Other authors indicate that clear and effective communication is fundamental to create changes in health organizations ^40^. In addition, a more informal or close communication style, based on closeness and personal interaction, can positively influence the collaboration and participation of team members in problem solving and decision-making ^41^. The effective communication of the PHC manager with staff linked to the unit allows adequate planning of health actions, establishing priorities of care and rational use of resources ^11^.

Regarding the attitudes identified, autonomy and flexibility are stated as essential to perform the role ^20^ ^,^ ^21^ because it contributes positively to the sustainability of health systems ^42^. Professionalism also stands out, described by the authors as commitment to the development of others, one’s own, and that of the profession, in addition to acting ethically and balancing with personal and professional life ^22^.

Among the limitations of this review, it is possible to point out the low number and low geographical diversity of the studies analyzed, since only two of them were conducted outside Brazil. Furthermore, this review has multiple designs included, so the articles provide very diverse information and results, which may make it difficult to synthesize these results in a single review. In addition, the low performance of some studies in the evaluation of methodological quality stands out, specifically in the item of sample representativeness regarding the target population. These factors, together, compromise the applicability of the results in other locations. For future research, the development of competency profiles that consider the specific environment in which PHC managers operate is suggested. Likewise, it is important to evaluate the self-perception of the required competencies, with the purpose to establish training and support programs to strengthen their management. Finally, primary care units, their management, and the professionals in charge may differ in each country. Therefore, the results should be applied with caution, considering local contexts and the type of professional in the management role. This is reinforced by Kakemam et al. ^43^, which underline the importance of adapting management competencies to local contexts in PHC. It highlights competencies should be relevant to particularities of each health system and the specific needs of the population, promoting more effective and contextualized management.

## Conclusions

PHC managers require training in the health area, as well as Administration knowledge and use of management indicators. Among the fundamental skills to perform their role, leadership, teamwork, training, communication, and the ability to recognize skills in others stand out, which, with a flexible and autonomous attitude, allow an adequate performance in the face of the diverse needs and challenges presented by PHC in the different contexts in which it operates.

## References

[1] Organización Mundial de la Salud (1978). Declaración de Alma Ata. Conferencia Internacional sobre Atención Primaria de Salud..

[2] World Health Organization (2019). Declaration of Astana: Global Conference on Primary Health Care.

[3] Murphy P, Burge F, Wong S (2019). Measurement and rural primary health care a scoping review. Rural Remote Health.

[4] Chotchoungchatchai S, Marshall AI, Witthayapipopsakul W, Panichkriangkrai W, Patcharanarumol W, Tangcharoensathien V (2020). Primary health care and sustainable development goals. Bull World Health Organ.

[5] Dois A, Bravo P, Contreras A, Soto MG, Mora I (2018). Formación y competencias para los equipos de atención primaria desde la mirada de expertos chilenos. Rev Panam Salud Pública.

[6] Organización Panamericana de la Salud (2019). Salud universal en el siglo XXI: 40 años de Alma-Ata. Informe de la Comisión de Alto Nivel.

[7] Chamberland-Rowe C, Simkin S, Bourgeault IL (2021). An integrated primary care workforce planning toolkit at the regional level (part 1) qualitative tools compiled for decision-makers in Toronto, Canada. Hum Resour Health.

[8] Douglas KA, Rayner FK, Yen LE, Wells RW, Glasgow NJ, Humphreys JS (2009). Australia's primary health care workforce - research informing policy. Med J Aust.

[9] Organización Panamericana de la Salud (2005). Llamado a la Acción de Toronto: 2006-2015. Hacia una década de recursos humanos en salud para las américas. Reunión regional de los observatorios de recursos humanos en salud.

[10] Chouhan VS, Srivastava S (2014). Understanding competencies and competency modeling - a literature survey. IOSR Journal of Business and Management.

[11] Organización Panamericana de la Salud (2007). Renovación de la atención primaria de salud en las Américas: documento de posición de la Organización Panamericana de la Salud/Organización Mundial de la Salud (OPS/OMS).

[12] Kyei N, Agyepong IA, van Dijk H, Kwamie A (2019). Facility management associated with improved primary health care outcomes in Ghana. PLoS One.

[13] Gilson L, Elloker S, Olckers P, Lehmann U (2014). Advancing the application of systems thinking in health South African examples of a leadership of sensemaking for primary health care. Health Res Policy Syst.

[14] Puertas EB, Sotelo JM, Ramos G (2020). Liderazgo y gestión estratégica en atención primaria de salud. Rev Panam Salud Pública.

[15] Fernandes JC, Cordeiro BC, Rezende AC, Freitas DS (2019). Competências necessárias ao gestor de unidade de saúde da família um recorte da prática do enfermeiro. Saúde Debate.

[16] Nieuwboer MS, van der Sande R.van der Marck MA.Olde Rikkert MGM.Perry M (2019). Clinical leadership and integrated primary care a systematic literature review. Eur J Gen Pract.

[17] Page MJ, McKenzie JE, Bossuyt PM, Boutron I, Hoffmann TC, Mulrow CD (2021). The PRISMA 2020 statement an updated guideline for reporting systematic reviews. BMJ.

[18] Ouzzani M, Hammady H, Fedorowicz Z, Elmagarmid A (2016). Rayyan - a web and mobile app for systematic reviews. Syst Rev.

[19] Hong QN, Pluye P, Fàbregues S, Bartlett G, Boardman F, Cargo M (2018). Mixed Methods Appraisal Tool (MMAT), version 2018. Education for Information.

[20] Fernandes JC, Cordeiro BC (2019). Gerência de unidade básica de saúde discutindo competências gerenciais com o enfermeiro gerente. Rev APS.

[21] Farah BF, Dutra HS, Sanhudo NF, Costa LM (2017). Percepção de enfermeiros supervisores sobre liderança na atenção primária. Rev Cuid (Bucaramanga).

[22] Dorji K, Tejativaddhana P, Siripornpibul T, Cruickshank M, Briggs D (2019). Leadership and management competencies required for Bhutanese primary health care managers in reforming the district health system. J Healthc Leadersh.

[23] Dikic M, Nikolic D, Todorovic J, Terzic-Supic Z, Kostadinovic M, Babic U (2020). Alignment of perceived competencies and perceived job tasks among primary care managers. Healthcare (Basel).

[24] Silva LB, Sousa MHO, Íñiguez-Rueda L (2022). Managers' views on professional competencies for primary health care. Sage Open.

[25] André AM, Ciampone MHT, Santelle O (2013). Tendências de gerenciamento de unidades de saúde e de pessoas. Rev Saúde Pública.

[26] Lopes AG, Narattharaksa K, Siripornpibul T, Briggs D (2020). An assessment of management competencies for primary health care managers in Timor-Leste. Int J Health Plann Manage.

[27] Appiah-Agyekum NN, Kayi EA, Appiah-Agyekum J, Tetteh Nyanyofio JG, Otoo DD (2022). Capacity issues in primary health care implementation examples from Ghana. Health Educ.

[28] Moosa S, Derese A, Peersman W (2017). Insights of health district managers on the implementation of primary health care outreach teams in Johannesburg, South Africa a descriptive study with focus group discussions. Hum Resour Health.

[29] Lowen IMV, Peres AM, Crozeta K, Bernardino E, Beck CLC (2015). Managerial nursing competencies in the expansion of the Family Health Strategy. Rev Esc Enferm USP.

[30] Munyewende PO, Levin J, Rispel LC (2016). An evaluation of the competencies of primary health care clinic nursing managers in two South African provinces. Glob Health Action.

[31] Assad SGB, Valente GSC, Santos SCP, Cortez EA (2021). Training and practice of nurses in primary care management perspectives of Schön's Theory. Rev Bras Enferm.

[32] Acevedo Ayala J, Lazo Pérez MA, Ávila Sánchez M (2023). Perfil directivo en la atención primaria de salud en Chile. Revista Cubana de Tecnología de la Salud.

[33] Flessa S, De Allegri M (2022). Healthcare management and health economics. Healthcare (Basel).

[34] Armijos JC, Núñez Mondaca A (2020). Indicadores de gestión para evaluar el desempeño de hospitales públicos un caso de estudio en Chile y Ecuador. Rev Med Chil.

[35] Skagerström J, Fernemark H, Nilsen P, Seing I, Hårdstedt M, Karlsson E (2023). Challenges of primary health care leadership during the COVID-19 pandemic in Sweden a qualitative study of managers' experiences. Leadership Health Serv (Bradf).

[36] True G, Stewart GL, Lampman M, Pelak M, Solimeo SL (2014). Teamwork and delegation in medical homes primary care staff perspectives in the Veterans Health Administration. J Gen Intern Med.

[37] Eddy K, Jordan Z, Stephenson M (2016). Health professionals' experience of teamwork education in acute hospital settings. JBI Database System Rev Implement Rep.

[38] Buljac-Samardzic M, Doekhie KD, van Wijngaarden JDH (2020). Interventions to improve team effectiveness within health care a systematic review of the past decade. Hum Resour Health.

[39] Rabkin SW, Frein M (2021). Overcoming obstacles to develop high-performance teams involving physician in health care organizations. Healthcare (Basel).

[40] Liang Z, Howard PF, Koh LC, Leggat S (2013). Competency requirements for middle and senior managers in community health services. Aust J Prim Health.

[41] Fanelli S, Lanza G, Enna C, Zangrandi A (2020). Managerial competences in public organisations the healthcare professionals' perspective. BMC Health Serv Res.

[42] Lennox L, Maher L, Reed J (2018). Navigating the sustainability landscape a systematic review of sustainability approaches in healthcare. Implement Sci.

[43] Kakemam E, Dargahi H, Tourani S, Djalalinia S, Mosavi M, Goharinezhad S (2023). Exploring the challenges of the healthcare system in Iran a qualitative study. BMC Health Serv Res.

